# Recitation Mode Is Associated With Salivary CREB and Cortisol Responses during Qur'anic Recitation

**DOI:** 10.1002/brb3.71674

**Published:** 2026-08-16

**Authors:** Süheyb Okur, Yusuf Topyay, Bülent Bayraktar

**Affiliations:** ^1^ Faculty of Theology Department of Psychology of Religion Bayburt University Bayburt Türkiye; ^2^ Faculty of Theology Department of Basic Islamic Sciences Bayburt University Bayburt Türkiye; ^3^ Faculty of Health Sciences Department of Physiotherapy and Rehabilitation Bayburt University Bayburt Türkiye

**Keywords:** cortisol, CREB, memorization, Qur'an, recitation, salivary biomarkers

## Abstract

**Background:**

Spiritual practices such as Qur'anic recitation are commonly associated with psychological and physiological regulation; however, their salivary biomarker correlates remain insufficiently understood. This exploratory study examined whether different modes of Qur'anic recitation were associated with changes in salivary cAMP response element‐binding protein (CREB), a biomarker related to plasticity‐associated pathways, and cortisol, a marker of hypothalamic‐pituitary‐adrenal axis activity.

**Methods:**

The sample comprised three nonrandomized, criterion‐defined conditions: Passive Control (*n* = 20; participants unable to read Qur'anic Arabic), Sight‐Reading (*n* = 30), and Memorized Recitation (*n* = 30). Saliva samples were collected immediately before and after the experimental task or passive rest period. Salivary CREB and cortisol concentrations were quantified using ELISA. Post‐task CREB levels were analyzed using ANCOVA with baseline CREB as a covariate. Cortisol responses were analyzed using change scores, Welch's ANOVA, and an additional robust factorial model with HC3 heteroscedasticity‐consistent standard errors to evaluate exploratory Condition × Gender patterns.

**Results:**

After controlling for baseline CREB levels, experimental condition had a significant effect on post‐task salivary CREB, *F*(2, 76) = 8.16, *p* < 0.001, *η* ^2^ *p* = 0.18. Memorized recitation showed higher adjusted post‐task CREB levels than both sight‐reading and passive control conditions. Cortisol change scores also differed significantly across conditions, Welch's *F*(2, 40.02) = 30.76, *p* < 0.001. Both active recitation conditions were associated with cortisol reduction relative to passive rest, with no significant difference between sight‐reading and memorized recitation. Although descriptive data suggested a possible gender‐related divergence in the memorized recitation condition, the Condition × Gender interaction did not remain statistically significant under robust estimation and was therefore interpreted as exploratory.

**Conclusion:**

These preliminary findings suggest that Qur'anic recitation modes are associated with distinct salivary biomarker patterns. Memorized recitation was linked to higher adjusted salivary CREB levels, whereas both active recitation modes were associated with cortisol attenuation relative to passive rest. Because the present design did not include active non‐Qur'anic vocalization or neutral reading control conditions, the specificity of these effects to Qur'anic content cannot be determined. Future studies should include active control conditions and larger gender‐stratified samples to clarify the mechanisms underlying these biomarker responses.

## Graphical abstract

The study compared three conditions: passive rest, sight‐reading, and memorized Qur'anic recitation. Memorized recitation was associated with higher adjusted post‐task salivary CREB levels than sight‐reading and passive rest, whereas both active recitation conditions were associated with cortisol attenuation relative to passive rest. Gender‐related cortisol patterns were interpreted as exploratory, and the specificity of these effects to Qur'anic content cannot be determined because active non‐Qur'anic vocalization or neutral reading controls were not included.

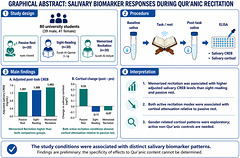

## 1 Introduction

The human brain, as the central regulatory system of the nervous system, supports not only basic physiological functions but also higher‐order processes such as attention, executive control, learning, and memory consolidation (Kandel et al. 2021). Learning can be understood as the acquisition and organization of new information, skills, and behavioral patterns, and it is closely linked to the processes of encoding, consolidation, and retrieval (Schunk 2012). Within Islamic educational practice, Qur'anic memorization has also been discussed as a structured learning activity involving repetition, reinforcement, and sustained cognitive engagement (Saat et al. 2011).

A growing body of research has examined the psychological, spiritual, and physiological effects of listening to, reciting, or memorizing the Qur'an. Systematic and scoping reviews suggest that Qur'anic recitation and listening have been associated with favorable outcomes in anxiety, stress, depression, sleep, quality of life, and selected physiological parameters (Che Wan Mohd Rozali et al. 2022; Moulaei et al. 2023). Reviews focused on neural correlates further indicate that Qur'anic listening studies have commonly used EEG or MEG outcomes, particularly alpha and theta activity, although methodological heterogeneity and risk of bias remain important limitations (Kannan et al. 2022; Majidi and Rajabi‐Tavakkol 2025). Music‐based therapeutic interventions and group chanting have also been examined in relation to psychological and physiological outcomes (Almerud and Petersson 2003; Perry et al. 2024); therefore, interpretations of Qur'anic recitation should consider both its specific spiritual meaning and the broader acoustic, rhythmic, vocal, and attentional features shared with other contemplative vocal practices.

From a neurocognitive perspective, Qur'anic recitation with tartil may involve several partially overlapping processes: rhythmic auditory stimulation, controlled articulation, auditory feedback, sustained attention, phonological sequencing, and memory retrieval. Rhythmic auditory stimulation is often discussed through the concept of neural entrainment, although recent work emphasizes that entrainment should be used cautiously because phase‐locked neural responses may arise from several mechanisms that are difficult to dissociate experimentally (Duecker et al. 2024). In addition, spoken recitation requires the integration of speech perception and speech production systems, including auditory‐motor feedback loops that support real‐time vocal control (Arjmandi and Behroozmand 2024). Recitation and comprehension also depend on working‐memory resources for maintaining phonological and semantic information during language processing (Martin et al. 2025).

Earlier Qur'an‐related studies have examined diverse outcomes, including hifz and brain‐related learning processes, cognitive or memory performance, anxiety reduction, EEG responses, short‐term memory, blood pressure, psychoacoustic responses, and psychological well‐being (Abdullah and Omar 2011; Fauzan and Abidin 2017; Kazemi et al. 2004; Kutlu et al. 2019; Novembrina et al. 2021; Rahman et al. 2018; Samhani et al. 2019; Saquib et al. 2017; Suteja Putra et al. 2018; Wahyuni et al. 2021). Although these studies have contributed to the field, many rely on self‐report measures, small samples, heterogeneous designs, or passive control conditions. Therefore, additional biomarker‐based studies are needed to clarify how different modes of Qur'anic practice are associated with measurable physiological responses.

The memorization and accurate recitation of the Qur'an, particularly hifz, represents a disciplined cognitive practice that requires repeated encoding, long‐term storage, and effortful retrieval of precisely ordered verbal material. Prior work has linked Qur'an memorization to later academic achievement and event‐related potential features, suggesting that memorization may involve measurable cognitive and electrophysiological correlates (Akbari et al. 2021; Nawaz and Jahangir 2015). Reading aloud requires visual processing, phonological decoding, and articulation (Kurudayıoğlu 2011); accurate Qur'anic recitation additionally requires controlled pronunciation and adherence to tajwid rules (Nelson 2001). Lifelong engagement in cognitively demanding activities has been associated with cognitive reserve and reduced risk of cognitive decline (Wang et al. 2012), while the religious value of literal Qur'anic reading underscores the ecological relevance of studying recitation as a lived scriptural practice (Dağdeviren 2017).

The distinction between sight‐reading and memorized recitation is also theoretically relevant to retrieval‐practice research. Retrieval practice, compared with repeated study or passive re‐exposure, is associated with enhanced long‐term memory and with neural activity in temporal, frontal, prefrontal, parietal, and striatal networks (Guran et al. 2022; Marin‐Garcia et al. 2021). Retrieval practice may also facilitate memory updating by modifying medial prefrontal representations (Ye et al. 2020). These findings suggest that reciting from memory may impose greater retrieval‐related demands than reading from text, although this possibility requires direct empirical testing within Qur'anic recitation contexts.

At the molecular level, neuroplasticity refers to the nervous system's capacity for structural and functional adaptation in response to environmental demands, learning, and injury (Marzola et al. 2023; Tataranu and Rizea 2025). cAMP response element‐binding protein (CREB) is a transcription factor implicated in synaptic plasticity, neurogenesis, neuronal survival, and memory‐related processes (Amidfar et al. 2020; Sharma and Singh 2020; Silva et al. 1998). In the present study, CREB was measured in saliva; therefore, it is treated as a peripheral biomarker related to CREB‐associated biological pathways rather than as a direct measure of central nervous system plasticity.

Physiological stress responses are strongly linked to cortisol, a glucocorticoid hormone regulated by the hypothalamic‐pituitary‐adrenal (HPA) axis (Herman et al. 2016; Moyers and Hagger 2023; Stewart and Krone 2011). Cortisol is adaptive in acute stress regulation but may have complex effects on cognition, emotion, and memory when dysregulated or persistently elevated. Because recitation practices may involve vocalization, rhythmic breathing, attentional focus, and affective meaning, cortisol provides a useful biomarker for examining whether different recitation modes are associated with acute stress‐related physiological change.

Recent biomarker‐based studies of Qur'anic recitation have begun to move beyond subjective self‐report outcomes by examining neurotrophic and stress‐related salivary markers, including BDNF, apelin, NDNF, cortisol, and monoamines (Okur and Bayraktar 2026; Okur and Bayraktar 2026). Nevertheless, the specific salivary biomarker patterns associated with sight‐reading versus memorized recitation remain insufficiently understood. Moreover, because active non‐Qur'anic vocalization or neutral reading control conditions are often absent from this literature, caution is required when interpreting whether observed effects are specific to Qur'anic content or reflect broader mechanisms such as vocalization, rhythm, breathing, attention, familiarity, or meaningful language.

Previous studies by our research group addressed related but scientifically distinct questions. Two cross‐sectional studies examined associations between metacognitive beliefs and single‐time‐point salivary biomarkers: one assessed CREB and cortisol, whereas the other assessed cortisol, BDNF, and NDNF (Okur and Bayraktar 2025; Okur et al. 2025). Neither study included Qur'anic recitation, an experimental task, or pre–post measurements. Two subsequent controlled studies compared sight‐reading from the Mushaf with passive rest and focused, respectively, on BDNF, apelin, and cortisol and on NDNF, serotonin, dopamine, and cortisol (Okur and Bayraktar 2026; Okur and Bayraktar 2026). However, neither compared sight‐reading with memorized recitation or examined acute salivary CREB responses to recitation. The present study addresses this gap by comparing passive rest, sight‐reading, and memorized recitation within a single pre–post framework.

Accordingly, the present exploratory study examined whether different modes of Qur'anic recitation were associated with changes in salivary CREB and cortisol. The study compared passive rest, sight‐reading from the Qur'anic text, and memorized recitation. We hypothesized that memorized recitation would be associated with higher adjusted post‐task salivary CREB levels than sight‐reading and passive rest, reflecting the additional retrieval‐related demands of reciting from memory. We also hypothesized that both active recitation conditions would be associated with cortisol attenuation relative to passive rest. Finally, because stress and cognitive‐load responses may vary by gender, we explored whether cortisol change scores differed as a function of Condition × Gender, while treating these gender‐stratified analyses as exploratory.

## 2 Method

This research was designed as an exploratory comparative study to investigate the neurobiological and endocrinological effects of reciting the Holy Qur'an with tartil. Prior to data collection, ethical approval was obtained from the Bayburt University Rectorate Ethics Committee. The study population consists of students from the Faculty of Theology at Bayburt University, selected on a voluntary basis. The sample comprises 80 students (39 male, 41 female) aged 18–25 who agreed to participate. Selecting university students as the sample rests on several primary justifications. First, the 18–25 age range forms a relatively homogeneous group regarding cognitive functions and hormonal balance. This homogeneity may help minimize the impact of confounding variables such as age, potentially allowing for a clearer observation of recitation's net effect on the biomarkers. Second, university students engaged in active learning and memorization processes offer a highly suitable population for a study requiring the activation of memory mechanisms. Finally, the practical accessibility of students within Bayburt University ensured the logistical feasibility of the research.

### 2.1 Relationship to Previous Publications and Data Provenance

The present study was derived from the first author's doctoral dissertation and was conducted under ethical approval dated February 7, 2023 (Decision No. 33), preceding the authors’ subsequently approved studies involving metacognition or Qur'anic recitation. The doctoral research constituted a separate project with independent participant recruitment, informed consent, saliva collection, laboratory measurement, data entry, and statistical analysis. No participants, saliva samples or aliquots, biomarker measurements, raw ELISA absorbance values, or analytical data records were shared with or reused in the related publications (Okur and Bayraktar 2026; Okur and Bayraktar 2026; Okur and Bayraktar 2025; Okur et al. 2025). Although cortisol was included as an HPA‐axis marker in several studies, and one separate cross‐sectional study also measured CREB, all measurements originated from independently recruited participants and separately collected and assayed biological samples.

Given the specific criteria required regarding the ability to recite the Qur'an, the study included undergraduate students across the first, second, third, and fourth years of study. Accordingly, criterion sampling ‐a purposive sampling technique‐ was employed for participant selection. To examine the relationship between the brain and the act of recitation, the parameters were limited to the CREB protein and the Cortisol hormone. The research does not include any examination aimed at comprehending the semantic dimension of the verses; the focus remains strictly on the phonetic articulation of the Arabic text in accordance with recitation rules (tartil).

Within the study design, participants were divided into three main groups based on the type of recitation task: Sight‐Reading Group (*n* = 30), Memorized Recitation Group (*n* = 30), and Passive Control Group (*n* = 20). The Passive Control Group consisted of students lacking the ability to read the Qur'an in Arabic (non‐reciters); these individuals were instructed to rest with their eyes closed in a quiet, isolated environment for the duration of the experiment. Participants were not randomly assigned to the three conditions. Condition membership was determined by Qur'anic reading ability and eligibility for the designated sight‐reading, memorized‐recitation, or non‐reciter passive‐control task.

A commission of subject‐matter academic staff selected the surahs used in the active recitation tasks. Following their evaluations, Surah Al‐Qamar (verses 7–16) was selected for the sight‐reading task, and Surah Al‐Adiyat was designated for the memorized recitation task. The primary reference point for choosing these specific texts is that both are largely equivalent in terms of phonetic load. This equivalency is based on the articulatory density of letters unique to Arabic (e.g., ‘dat’[ض] and ‘za’[ظ]) and the frequency of tajwid rules (such as idgham ma'al ghunnah, ikhfa, and qalqalah). The methodological rationale for not using the same text for both sight‐reading and memorized recitation tasks stems from the students’ academic curriculum. The sight‐reading practices and the memorization (hifz) curriculum involved in the academic formation of theology students differ. To preserve the study's ecological validity, two separate surahs—aligned with the students’ curricular habits yet tentatively standardized for phonetic difficulty—were selected for each recitation method.

#### 2.1.1 Procedure and Sample Collection

Each participant in the active reading groups (Sight‐Reading and Memorized Recitation) was instructed to perform their designated recitation task. Before and immediately after the recitation session, saliva samples were collected from the participants using Salivette tubes. In contrast, individuals in the Passive Control Group were instructed to rest for 10 min with their eyes closed in the same sound‐isolated room where the active recitations took place. For these non‐reciters, saliva samples were similarly collected before and after this designated rest period. Upon completion of the collection phase, all biological samples were stored at ‐20°C until laboratory analysis.

#### 2.1.2 Data Collection Tools

Prior to the recitation sessions, participants were administered a Personal Information Form alongside the Spirituality Scale. Developed by Şirin, this scale is designed to assess various dimensions of human spirituality in adolescents and adults. The instrument consists of 27 items and utilizes a 5‐point Likert‐type rating system, with response options ranging from (1) “Not at all applicable to me” to (5) “Totally applicable to me.” The Spirituality Scale encompasses seven sub‐dimensions: Spiritual Coping, Transcendence, Spiritual Experience, Search for Meaning, Spiritual Contentment, Connectedness, and Harmony with Nature. The scale was selected because its multidimensional structure includes domains directly relevant to the present study, particularly Transcendence, Search for Meaning, and Spiritual Contentment. In the original validation study, Şirin reported evidence supporting the construct validity and internal consistency of the scale (Şirin 2018). While the original validation study reported a high internal consistency coefficient of 0.90, the Cronbach's alpha reliability coefficient for the current study sample was calculated as 0.89.

#### 2.1.3 Measures and Biomarker Acquisition

Demographic information and responses to the Spirituality Scale were obtained through structured face‐to‐face interviews. To examine salivary biomarker responses, salivary CREB and cortisol were selected as the primary biological markers. To control for diurnal fluctuations in biomarker concentrations, all saliva samples were consistently collected between 08:00 and 09:00. Unstimulated whole saliva samples were collected by direct expectoration into Salivette collection tubes, without use of the swab insert, immediately before (baseline) and immediately after (post‐task) the assigned recitation or passive‐rest procedure.

To reduce the risk of sample contamination, participants were instructed to abstain from consuming food or beverages, brushing their teeth, and ingesting dietary components that could alter biomarker concentrations for at least 1 h before sample collection. Immediately after collection, samples were centrifuged at 2000 × *g* for 20 min in a refrigerated centrifuge (NF 1200R, Turkey). The supernatant was aliquoted and stored at −20°C until biochemical analysis.

Biochemical Assessment of Salivary CREB and Cortisol: All targeted biomarker concentrations were quantified using human‐specific Sandwich Enzyme‐Linked Immunosorbent Assay (ELISA) kits, adhering strictly to the manufacturers’ protocols. The specific analytical kits, catalog numbers, assay ranges, and corresponding manufacturer‐reported sensitivities utilized in this study are detailed below:

‐ Human cAMP Response Element‐Binding Protein (CREB) ELISA Kit: SinoGeneclon, Cat. No. SG‐11988; assay range: 0.625–20 ng/mL; sensitivity: 0.078 ng/mL. Assays were performed using sample volumes of 50–100 µL, and optical density was read at 450 nm to quantify salivary CREB concentrations.
‐ Human Cortisol ELISA Kit: SinoGeneclon, Cat. No. SG‐00111; manufacturer‐reported detection range: 5–100 µg/L; sensitivity: 0.02 µg/L.

The manufacturer's calibrator concentrations and reported assay range were specified in µg/L. Sample concentrations calculated from the ELISA calibration curve were initially obtained in µg/L and were converted to µg/dL by dividing by 10 before statistical analysis (1 µg/dL = 10 µg/L). Accordingly, all absolute cortisol concentrations and cortisol change scores reported in this manuscript are expressed in µg/dL.

#### 2.1.4 Statistical Analysis

Analyses were conducted using IBM SPSS Statistics (Version 27.0). The analytical strategy reflected the exploratory design of the study. Participants were analyzed across three study conditions: the Sight‐Reading Group (*n* = 30), the Memorized Recitation Group (*n* = 30), and the Passive Control Group (*n* = 20).

For salivary CREB, preliminary analyses confirmed compliance with the necessary parametric assumptions, including the homogeneity of regression slopes. Accordingly, a one‐way ANCOVA was used to examine post‐task CREB levels across experimental conditions while controlling for baseline CREB. When the omnibus condition effect was significant in the CREB ANCOVA, Bonferroni‐adjusted pairwise comparisons were conducted. To explore possible gender‐related patterns, a 3 (Condition) × 2 (Gender) ANCOVA was additionally conducted with baseline CREB as the covariate.

To formally evaluate the baseline CREB imbalance, baseline concentrations were compared across conditions. Because Levene's test indicated unequal variances, Welch's one‐way ANOVA was used, followed by Games–Howell pairwise comparisons. As a sensitivity analysis, individual CREB change scores were calculated as post‐task minus baseline CREB. The change scores were likewise evaluated using Welch's ANOVA and Games–Howell comparisons because their variances differed across conditions.

For cortisol, the assumption of homogeneity of regression slopes was violated; therefore, cortisol change scores were calculated by subtracting baseline cortisol from post‐task cortisol. Because Levene's test indicated unequal variances across the experimental conditions, Welch's ANOVA was used for the main condition effect, followed by Tamhane's T2 post hoc comparisons. To examine exploratory Condition × Gender patterns in cortisol change scores, a 3 × 2 factorial model was initially considered. However, because Levene's test also indicated unequal variances across the Condition × Gender cells, the factorial model was re‐estimated using HC3 heteroscedasticity‐consistent robust standard errors. Given the small cell sizes in the gender‐stratified analyses, these findings were interpreted as exploratory.

Finally, exploratory Pearson product–moment correlations were used to examine bivariate associations between salivary CREB, cortisol, and Spirituality Scale total and subdimension scores. Correlation analyses were two‐tailed and interpreted descriptively because of the exploratory nature of the study and the number of bivariate comparisons. The alpha level was set at 0.05 for inferential tests. Because the correlation analyses were exploratory and involved multiple bivariate comparisons, these results were interpreted descriptively rather than as confirmatory evidence.

## 3 Results

This section presents the findings derived from the salivary CREB and Cortisol samples collected from the study cohort (*N* = 80). Within the consolidated comparative framework, the data reflect two primary measurement phases: baseline levels (collected prior to any intervention) and post‐task levels (collected immediately following the respective sight‐reading, memorized recitation, or resting control conditions).

Prior to conducting the primary inferential analyses, the data were examined for outliers, distributional characteristics, and assumptions relevant to each model. Because assumption checks differed across biomarkers, the analytical approach was tailored accordingly: ANCOVA was retained for salivary CREB when the homogeneity of regression slopes assumption was met, whereas cortisol was analyzed using change scores and robust procedures when model assumptions were violated.

To provide a transparent account of participants’ initial physiological states, absolute baseline salivary CREB and cortisol values for each experimental condition are reported in Table S1.

Baseline CREB differed significantly across conditions. The homogeneity‐of‐variance assumption was violated, Levene's *F*(2, 77) = 16.03, *p* < 0.001; therefore, Welch's ANOVA was used. The omnibus test was significant, Welch's *F*(2, 42.95) = 41.47, *p* < 0.001. Games–Howell comparisons showed that baseline CREB was lower in the Passive Control condition (*M* = 0.580, SD = 0.107 ng/mL) than in both the Memorized Recitation condition (*M* = 1.345, SD = 0.582 ng/mL, *p* < 0.001) and the Sight‐Reading condition (*M* = 1.149, SD = 0.477 ng/mL, *p* < 0.001). Baseline CREB did not differ significantly between the Memorized Recitation and Sight‐Reading conditions (*p* = 0.334).

The distribution of the study cohort across the three experimental conditions is presented in Table 1. To investigate the effects of different Qur'anic recitation methods on post‐task salivary CREB levels while controlling for baseline physiological variation, a one‐way ANCOVA was performed. Preliminary checks confirmed that the data met the assumption of homogeneity of regression slopes, F(2, 74) = 1.97, p = 0.147, supporting the use of baseline CREB as a covariate. As detailed in Table 2, baseline CREB was a significant predictor of post‐task levels, F(1, 76) = 1169.25, p < 0.001, η²p = 0.94. After adjustment for baseline values, a statistically significant main effect of experimental condition was observed, F(2, 76) = 8.16, p < 0.001, η²p = 0.18.

**TABLE 1:** Distribution of the study cohort.

Groups	*n*
Sight‐Reading Group	30
Memorized Recitation Group	30
Passive Control Group	20

**TABLE 2:** ANCOVA results for salivary CREB levels across experimental conditions.

Condition	*n*	Observed mean (SD)	Adjusted mean (*M*_adj_)	SE
Passive Control	20	0.62 (0.10)	1.287^a^	0.040
Sight‐Reading	30	1.46 (0.60)	1.368^a^	0.029
Memorized Recitation	30	1.84 (0.83)	1.493^b^	0.031

*Note: N* = 80. Adjusted means are predicted at the mean value of the covariate (baseline CREB = 1.0802). Means with different superscript letters (^a^,^b^) represent statistically significant differences at the *p* < 0.05 level based on Bonferroni post hoc comparisons.

Bonferroni‐adjusted post hoc comparisons indicated that the adjusted mean for the Memorized Recitation Group (*M*_adj_ = 1.49, SE = 0.03) was significantly higher than both the Passive Control Group (*M*_adj_ = 1.29, SE = 0.04, *p* < 0.001) and the Sight‐Reading Group (*M*_adj_ = 1.37, SE = 0.03, *p* = 0.011). The difference between the Sight‐Reading and Passive Control Groups was not statistically significant (*p* = 0.342). These findings indicate that, after controlling for baseline CREB levels, memorized recitation was associated with higher adjusted post‐task salivary CREB levels than sight‐reading and passive rest.

A sensitivity analysis based on CREB change scores (post‐task minus baseline) also showed a significant condition effect (see Table S2). Mean CREB change was 0.043 ng/mL (SD = 0.038) in the Passive Control condition, 0.310 ng/mL (SD = 0.145) in the Sight‐Reading condition, and 0.500 ng/mL (SD = 0.324) in the Memorized Recitation condition. Because change‐score variances were unequal, Levene's *F*(2, 77) = 41.85, *p* < 0.001, Welch's ANOVA was retained, Welch's *F*(2, 42.89) = 71.22, *p* < 0.001. Games–Howell comparisons indicated greater CREB increases in both the Memorized Recitation and Sight‐Reading conditions than in Passive Control (both *p* < 0.001); the increase was also greater for Memorized Recitation than for Sight‐Reading (mean difference = 0.189 ng/mL, 95% CI [0.032, 0.347], *p* = 0.015). Thus, the sensitivity analysis confirmed the significant overall condition effect and the ordering of the group means. However, unlike the baseline‐adjusted ANCOVA, it also identified a significant Sight‐Reading versus Passive Control difference.

To explore whether salivary CREB responses differed by gender, a 3 (Condition) × 2 (Gender) ANCOVA was conducted on post‐task CREB levels, with baseline CREB included as a covariate. Descriptive and adjusted means are presented in Table 3. Because the Condition × Gender cells ranged from n = 10 to n = 16, these gender‐stratified analyses were interpreted as exploratory. Condition‐specific cortisol change scores are presented in Table 4.

**TABLE 3:** Exploratory two‐way ANCOVA results for salivary CREB levels by condition and gender.

Condition	Gender	*n*	Observed mean (SD)	Adjusted mean (*M*_adj_)	SE
Passive Control	Male	10	0.59 (0.11)	1.289	0.055
	Female	10	0.65 (0.09)	1.285	0.054
Sight‐Reading	Male	15	1.47 (0.55)	1.387	0.042
	Female	15	1.45 (0.68)	1.349	0.042
Memorized Recitation	Male	14	1.82 (0.88)	1.505	0.044
	Female	16	1.87 (0.83)	1.482	0.042

*Note*: *N* = 80. Adjusted means are predicted at the mean value of the covariate, baseline CREB = 1.0802. Cell sizes ranged from *n* = 10 to *n* = 16; therefore, gender‐stratified comparisons were interpreted as exploratory.

**TABLE 4:** Welch's ANOVA results for modulations in salivary cortisol levels (change scores).

Condition	*n*	Mean ΔCortisol (µg/dL)	(SD)	SE
Passive Control	20	0.33^a^	0.23	0.05
Sight‐Reading	30	−0.11^b^	0.11	0.02
Memorized Recitation	30	−0.07^b^	0.17	0.03

*Note*: *N* = 80. The dependent variable is the cortisol change score, reported in µg/dL and calculated by subtracting baseline cortisol from post‐task cortisol. Positive values indicate an increase in cortisol, whereas negative values indicate a decrease. Means with different superscript letters (^a, b^) represent statistically significant differences at the *p* < 0.05 level based on Tamhane's T2 post hoc comparisons (assumptions of equal variances not met).

The analysis showed no significant main effect of gender on adjusted post‐task salivary CREB levels, *F*(1, 73) = 0.34, *p* = 0.561, *η* ^2^ *p* = 0.005. The Condition × Gender interaction was also not significant, *F*(2, 73) = 0.07, *p* = 0.936, *η* ^2^ *p* = 0.002. Thus, within the limits of the present sample, the higher adjusted salivary CREB levels observed in the memorized recitation condition did not appear to be moderated by gender.

Given the relatively small cell sizes, however, the absence of a significant interaction should not be interpreted as definitive evidence of gender‐independent CREB modulation. Rather, these findings suggest that the primary CREB‐related pattern in this sample was associated with recitation condition, particularly memorized recitation, while potential gender differences require confirmation in larger samples.

Welch's ANOVA revealed a significant main effect of experimental condition on cortisol change scores, Welch's *F*(2, 40.02) = 30.76, *p* < 0.001. Tamhane's T2 post hoc comparisons showed that both the Sight‐Reading Group and the Memorized Recitation Group differed significantly from the Passive Control Group, with both active recitation conditions showing cortisol reduction while the passive control condition showed a mean increase. The two active recitation conditions did not significantly differ from each other, *p* = 0.694.

These findings indicate that, within the present study design, both active Qur'anic recitation conditions were associated with cortisol attenuation relative to passive rest. However, because the study did not include an active non‐Qur'anic vocalization or neutral reading control condition, these results should not be interpreted as demonstrating effects specific to Qur'anic content.

To examine whether cortisol change scores varied as a function of condition and gender, a 3 (Condition) × 2 (Gender) factorial model was evaluated. Descriptive statistics are presented in Table 5. Cell sizes ranged from *n* = 10 to *n* = 16, indicating that gender‐stratified comparisons should be interpreted cautiously. In addition, Levene's test indicated a violation of the homogeneity of variance assumption across the Condition × Gender cells, *F*(5, 74) = 3.03, *p* = 0.015. Therefore, the factorial model was re‐estimated using HC3 heteroscedasticity‐consistent robust standard errors.

**TABLE 5:** Cortisol change scores by condition and gender and robust factorial test.

Panel A. Descriptive statistics for cortisol change scores					
Condition	Gender	*n*	Mean ΔCortisol (µg/dL)	SD	SE
Passive Control	Male	10	0.274	0.161	0.051
	Female	10	0.376	0.284	0.090
Sight‐Reading	Male	15	−0.090	0.125	0.032
	Female	15	−0.130	0.081	0.021
Memorized Recitation	Male	14	0.003	0.160	0.043
	Female	16	−0.142	0.144	0.036

Panel B. Robust factorial model for cortisol change scores				
Effect	df1	df2	Robust *F*	*p*
Condition	2	74	28.14	<0.001
Gender	1	74	3.49	0.066
Condition × Gender	2	74	2.33	0.104

*Note*: ΔCortisol was calculated as post‐task cortisol minus baseline cortisol and is reported in µg/dL. Negative values indicate cortisol reduction. Cell sizes ranged from *n* = 10 to *n* = 16; therefore, gender‐stratified findings were interpreted as exploratory. Robust *F* tests were estimated using HC3 heteroscedasticity‐consistent standard errors because Levene's test indicated unequal variances across the Condition × Gender cells, *F*(5, 74) = 3.03, *p* = 0.015.

The HC3 robust Type II model confirmed a significant main effect of condition on cortisol change scores, Robust *F*(2, 74) = 28.14, *p* < 0.001, indicating that cortisol trajectories differed across the passive control, sight‐reading, and memorized recitation conditions. The main effect of gender was not statistically significant, Robust *F*(1, 74) = 3.49, *p* = 0.066. Importantly, the Condition × Gender interaction did not remain statistically significant under robust estimation, Robust *F*(2, 74) = 2.33, *p* = 0.104.

Descriptively, both male and female participants in the Sight‐Reading condition showed cortisol reduction. In the memorized recitation condition, female participants showed a reduction in cortisol, whereas male participants showed little net change. However, because the robust Condition × Gender interaction was not statistically significant, this gender‐related pattern should be interpreted as exploratory and hypothesis‐generating rather than as evidence of a reliable gender‐specific moderation effect.

To explore bivariate associations between baseline salivary biomarker concentrations and self‐reported spirituality scores, a two‐tailed Pearson correlation analysis was conducted (see Table 6). Baseline CREB concentrations were negatively correlated with baseline cortisol concentrations (*r* = −0.412, *p* < 0.001), indicating that higher baseline salivary CREB concentrations tended to co‐occur with lower baseline cortisol concentrations in this sample.

**TABLE 6:** Relationships between baseline salivary CREB and cortisol concentrations and spirituality scale scores.

Variables	1	2	3	4	5	6	7	8	9	10
1. CREB	1									
2. Cortisol	−0.412**	1								
3. Spirituality (total)	0.215	−0.044	1							
4. Spiritual Coping	0.158	0.063	0.557**	1						
5. Transcendence	0.249*	−0.061	0.408**	0.557**	1					
6. Spiritual Experience	0.106	−0.001	0.783**	0.783**	0.408**	1				
7. Search for Meaning	0.228*	−0.118	0.427**	0.489**	0.523**	0.427**	1			
8. Spiritual Contentment	0.281*	−0.069	0.306**	0.505**	0.386**	0.306**	0.269*	1		
9. Connectedness	0.068	0.120	0.376**	0.549**	0.543**	0.376**	0.415**	0.403**	1	
10. Harmony with Nature	0.010	0.075	0.331**	0.184	0.116	0.331**	0.241*	0.052	0.042	1

*Note*: CREB and cortisol values represent baseline salivary concentrations measured before the experimental task or passive‐rest condition. **p < 0.01, *p < 0.05 (two‐tailed).

Baseline CREB concentrations showed weak positive associations with Spiritual Contentment (*r* = 0.281, *p* = 0.012), Transcendence (*r* = 0.249, *p* = 0.026), and Search for Meaning (*r* = 0.228, *p* = 0.042). Their association with the Total Spirituality score did not reach statistical significance (*r* = 0.215, *p* = 0.055). These correlations were interpreted as exploratory and should not be taken as evidence of causal relationships between trait‐level spirituality and baseline biomarker concentrations.

Baseline cortisol concentrations did not show significant linear associations with the Total Spirituality score or any of its subdimensions (all *p* > 0.05). Thus, trait‐level spirituality scores were not associated with pre‐task cortisol concentrations in this sample.

## 4 Discussion

The present exploratory study examined whether different modes of Qur'anic recitation were associated with changes in salivary CREB and cortisol levels. The main findings were threefold. First, after controlling for baseline CREB levels, memorized recitation was associated with higher adjusted post‐task salivary CREB levels than both sight‐reading and passive rest. Second, both active recitation conditions were associated with reductions in salivary cortisol relative to passive rest. Third, although descriptive cortisol patterns suggested a possible gender‐related divergence during memorized recitation, the Condition × Gender interaction did not remain statistically significant under HC3 robust estimation and should therefore be interpreted as exploratory. Together, these findings suggest that recitation mode may be associated with distinct salivary biomarker patterns, while also underscoring the need for caution in making content‐specific or mechanistic claims.

The present findings should also be distinguished from our previous reports. The earlier metacognition studies concerned cross‐sectional individual differences and did not involve Qur'anic recitation (Okur and Bayraktar 2025; Okur et al. 2025), whereas the previous recitation studies compared Mushaf reading with passive rest and focused on BDNF, apelin, NDNF, or monoaminergic biomarkers (Okur and Bayraktar 2026; Okur and Bayraktar 2026). The current study is the first within this research program to directly compare sight‐reading with memorized recitation and to examine acute salivary CREB responses to these recitation modes. The observed pattern—higher baseline‐adjusted post‐task CREB following memorized recitation but comparable cortisol attenuation across both active modes—suggests a potential distinction between retrieval‐sensitive and mode‐general biomarker responses. The present findings are not a reanalysis, subgroup report, or fragmented report of any previously published dataset.

The CREB change‐score sensitivity analysis reinforced the robustness of the overall condition effect and showed the largest mean increase during memorized recitation, followed by sight‐reading and passive rest. Nevertheless, the pairwise results were not identical across analytical approaches: sight‐reading differed from passive control in the change‐score analysis but not in the baseline‐adjusted ANCOVA. Accordingly, the overall association between recitation condition and CREB response appears robust, whereas the exact pairwise pattern—particularly the sight‐reading versus passive‐control contrast—should be regarded as model‐dependent and interpreted cautiously.

The higher adjusted post‐task salivary CREB levels observed in the memorized recitation condition may reflect the greater mnemonic and retrieval‐related demands of reciting from memory compared with reading from text. Retrieval‐practice studies indicate that active retrieval, compared with passive re‐exposure or restudy, strengthens long‐term memory and is associated with frontal, temporal, prefrontal, parietal, and striatal activity (Guran et al. 2022; Marin‐Garcia et al. 2021; Ye et al. 2020). In the present context, hifz requires the internal reconstruction of phonological sequences, verse order, rhythm, articulation rules, and recitation flow without continuous visual support. This interpretation is also consistent with broader neuroplasticity literature showing that learning and environmental demands can shape plasticity‐related biological processes (Marzola et al. 2023; Tataranu and Rizea 2025). However, CREB should be interpreted carefully: although CREB is central to memory‐ and plasticity‐related pathways (Amidfar et al. 2020; Sharma and Singh 2020; Silva et al. 1998), salivary CREB cannot by itself establish central synaptic remodeling, region‐specific brain activation, or direct neural plasticity.

The acoustic and rhythmic structure of tartil may also have contributed to the observed biomarker pattern. Structured auditory stimulation has been linked experimentally to BDNF and downstream CREB‐related pathways in animal models (Wang et al. 2023), and sustained engagement with music or structured auditory activity has been discussed as a potential contributor to cognitive reserve (Wolff et al. 2023). In Qur'anic contexts, recent biomarker studies have reported stress‐ and neurotrophic‐marker changes during recitation or rhythmic vocalization (Okur and Bayraktar 2026; Okur and Bayraktar 2026). Nevertheless, the current study measured salivary biomarkers rather than central nervous system activity. Therefore, the findings should be framed as peripheral biomarker responses associated with recitation mode, not as direct proof that memorized recitation maximizes neuroplastic activation in the brain.

A central interpretive issue is whether the observed biomarker changes are specific to Qur'anic recitation or reflect broader processes shared with other structured vocal practices. Reviews of Qur'anic listening have noted potential neural and psycho‐spiritual effects, including EEG changes in alpha and theta ranges, but they also emphasize heterogeneity in study quality and design (Kannan et al. 2022; Majidi and Rajabi‐Tavakkol 2025). More generally, chanting and other repetitive vocal practices may reduce cortisol and anxiety (Perry et al. 2024). Qur'anic recitation with tartil also involves rhythmic vocalization, paced breathing, sustained attention, auditory‐motor coordination, and phonological or semantic working‐memory demands, all of which may influence physiological regulation independently of Qur'anic content (Arjmandi and Behroozmand 2024, Martin et al. 2025; Duecker et al. 2024; Heyda et al. 2025; Morales‐Luque et al. 2025). Accordingly, the present findings should not be interpreted as isolating a Qur'an‐specific biological mechanism. Active control conditions involving neutral text reading, non‐Qur'anic vocalization, or matched rhythmic speech are needed to determine the degree to which the effects are content‐specific, rhythm‐related, breathing‐related, or retrieval‐related.

The cortisol findings indicate that both active recitation conditions were associated with cortisol attenuation relative to passive rest. This is consistent with the role of cortisol as a primary marker of HPA‐axis activity and stress regulation (Herman et al. 2016; Moyers and Hagger 2023; Stewart and Krone 2011). The reduction observed in both active conditions may be partly related to features common to recitation and other vocal practices, including rhythmic breathing, sustained vocalization, attentional focus, and auditory feedback. Studies of chanting indicate that repetitive vocal or silent practice can reduce cortisol and anxiety (Perry et al. 2024), while research on conscious breathing and voice‐autonomic links suggests plausible pathways through which vocal‐respiratory activity may modulate physiological arousal (Heyda et al. 2025; Morales‐Luque et al. 2025). Importantly, sight‐reading and memorized recitation did not differ significantly in cortisol reduction, suggesting that the stress‐related response observed here may be less dependent on memorized retrieval than the CREB‐related response. Because the Passive Control Group involved resting with eyes closed rather than an active matched task, however, the present design cannot determine whether cortisol attenuation was driven by Qur'anic content, vocalization, breathing rhythm, attentional engagement, familiarity, or the affective meaning of the practice.

The descriptive gender‐stratified cortisol pattern warrants cautious interpretation. In the conventional factorial analysis, the Condition × Gender interaction suggested a possible divergence during memorized recitation; however, this interaction did not remain statistically significant when the model was re‐estimated using HC3 heteroscedasticity‐consistent robust standard errors. Accordingly, the pattern—little net cortisol reduction among male participants during memorized recitation alongside continued reduction among female participants—should be treated as exploratory and hypothesis‐generating. One possible explanation is that memorized recitation may impose greater retrieval‐related effort for some participants, which could attenuate cortisol reduction. Prior work indicates that acute stress can affect working‐memory strategy use (Husa et al. 2023), neurocardiac stress signatures may vary by sex (Wriessnegger et al. 2025), working memory may predict HPA‐axis responses in males (Lin et al. 2020), and cortisol release under stress may relate differently to working‐memory performance in women and men (Luers et al. 2020). However, the present study did not directly measure cognitive load, perceived effort, task difficulty, performance anxiety, breathing rate, recitation accuracy, or working‐memory demand. Cognitive load should therefore be discussed only as a possible explanatory hypothesis, not as an established mechanism.

The inverse association between CREB and cortisol provides an additional exploratory perspective on the potential relationship between plasticity‐related and stress‐related biomarker responses. Neuroendocrine models indicate that acute stress and glucocorticoid activity can modulate synaptic plasticity mechanisms (dos‐Santos et al. 2023), and CREB‐related pathways are also implicated in memory and plasticity (Amidfar et al. 2020; Sharma and Singh 2020; Silva et al. 1998). In this sample, higher salivary CREB levels tended to co‐occur with lower cortisol levels. However, this correlation does not establish directionality or causation. It should be viewed as preliminary evidence that salivary CREB and cortisol may index partially different, and possibly interacting, physiological dimensions of recitation‐related response.

The correlations between salivary biomarkers and spirituality scores should also be interpreted cautiously. The Spirituality Scale used in this study was designed to assess multiple dimensions of spirituality, including Transcendence, Search for Meaning, and Spiritual Contentment (Şirin 2018). In the present data, CREB showed weak positive associations with selected spirituality dimensions, while cortisol showed no significant linear association with trait‐level spirituality. These findings should not be interpreted as evidence that spirituality causes CREB elevation or cortisol attenuation. Rather, they suggest that individual differences in spiritual orientation may be related to the broader psychological context in which recitation practices occur. Future research should examine whether state spiritual absorption, perceived meaning, emotional engagement, or attentional immersion mediate biomarker responses during recitation.

Overall, the present findings support a cautious interpretation: different Qur'anic recitation modes were associated with distinct salivary biomarker responses, with memorized recitation showing the highest adjusted salivary CREB levels and both active recitation modes showing cortisol attenuation relative to passive rest. These patterns are consistent with the possibility that mnemonic retrieval and structured vocal recitation engage partially distinct physiological pathways. However, the absence of active non‐Qur'anic and neutral reading controls prevents attribution of these effects specifically to Qur'anic content. The findings should therefore be viewed as preliminary evidence from an ecologically valid scriptural recitation context rather than as definitive proof of Qur'an‐specific neurobiological mechanisms.

### 4.1 Limitations and Future Directions

Several limitations should be acknowledged. First, the study included a Passive Control Group, which allowed comparison with a resting baseline; however, it did not include active control conditions. Therefore, the present design cannot determine whether the observed salivary CREB and cortisol patterns were specific to Qur'anic recitation or were instead related to more general features of the task, such as rhythmic vocalization, paced breathing, focused attention, phonological processing, memorized retrieval, emotional meaning, or familiarity with the material. Future studies should include active control groups, such as reading a neutral non‐rhythmic text aloud, reciting a rhythmically matched non‐Qur'anic Arabic text, silently reading matched material, or engaging in non‐Qur'anic vocalization matched for duration, rhythm, and respiratory demand.

Second, the Passive Control Group consisted of non‐reciters who rested with eyes closed. Although this design provided a useful resting baseline, it also introduced differences in Qur'anic reading ability, familiarity, and task engagement between the passive and active groups. Future designs should recruit participants capable of completing all task conditions and use within‐subject or randomized counterbalanced designs where feasible.

A related limitation is the substantial baseline imbalance in salivary CREB. Baseline concentrations were lower in the Passive Control condition than in both active recitation conditions. Because assignment was nonrandomized and partly determined by Qur'anic reading ability, this imbalance may reflect pre‐existing physiological or participant‐level differences. Although baseline CREB was included as a covariate in the primary ANCOVA, statistical adjustment cannot fully eliminate confounding arising from limited baseline comparability. Regression to the mean and model‐based extrapolation may also have influenced the adjusted estimates. The condition differences should therefore be interpreted as associations rather than causal effects of recitation mode.

Third, the gender‐stratified analyses were limited by small cell sizes, ranging from *n* = 10 to *n* = 16. In addition, the Condition × Gender interaction for cortisol did not remain statistically significant under robust estimation. Consequently, gender‐related cortisol patterns should be interpreted as exploratory and hypothesis‐generating. Future studies with larger and more balanced samples are needed to evaluate whether gender reliably moderates biomarker responses to recitation.

Fourth, cognitive load was not directly measured. Although memorized recitation plausibly involves greater retrieval‐related effort than sight‐reading, the study did not assess perceived task difficulty, mental effort, working memory demand, performance anxiety, recitation accuracy, breathing rate, or autonomic parameters such as heart rate variability. These measures should be incorporated in future studies to determine whether cognitive load or autonomic regulation mediates changes in salivary CREB and cortisol.

Fifth, salivary CREB should not be interpreted as a direct measure of central neuroplasticity. CREB is biologically relevant to plasticity‐related pathways, but the present study measured salivary concentrations rather than brain‐region‐specific CREB activity. Future research should combine salivary biomarkers with neuroimaging, EEG, neurotrophic markers, autonomic indices, and behavioral memory measures to clarify the relationship between peripheral biomarker changes and central neurocognitive processes.

Sixth, the sample consisted of healthy young theology students, which limits generalizability to older adults, clinical populations, non‐student samples, and individuals with different levels of recitation experience. The acute pre‐post design also prevents conclusions about long‐term effects of sustained Qur'anic recitation, hifz training, or spiritual practice on biomarker regulation. Longitudinal studies are needed to examine whether repeated practice produces stable changes in stress regulation, cognitive performance, or plasticity‐related biomarkers.

Finally, although all saliva samples were collected within a restricted morning window to reduce diurnal variability, future studies should more extensively control for factors that may influence cortisol and salivary biomarkers, including sleep quality, recent stress exposure, menstrual cycle phase, hormonal contraceptive use, caffeine intake, and prior physical activity.

## 5 Conclusion

This exploratory study provides preliminary evidence that different modes of Qur'anic recitation are associated with distinct salivary biomarker responses. After controlling for baseline CREB levels, memorized recitation was associated with higher adjusted post‐task salivary CREB levels than sight‐reading and passive rest. In contrast, both active recitation conditions were associated with cortisol attenuation relative to passive rest, with no significant difference between sight‐reading and memorized recitation. Descriptive gender‐related cortisol patterns were observed, but these did not remain statistically significant under robust estimation and should therefore be interpreted cautiously.

These findings suggest that memorized retrieval and active vocal recitation may relate to partially different biomarker profiles within an ecologically valid Qur'anic recitation context. However, because the study did not include active non‐Qur'anic vocalization or neutral reading control conditions, the specificity of these effects to Qur'anic content cannot be established. Future research using larger samples, active control tasks, direct cognitive‐load measures, autonomic indices, and neuroimaging or electrophysiological methods is needed to clarify the mechanisms linking recitation, stress regulation, and plasticity‐related biomarker responses.

## Author Contributions

**Bülent Bayraktar**: conceptualization, funding acquisition, project administration, software, formal analysis, supervision, data curation, resources, visualization, validation, methodology. **Süheyb Okur**: investigation, conceptualization, writing – original draft, writing – review and editing, formal analysis, software, project administration. **Yusuf Topyay**: writing – original draft, validation, visualization, writing – review and editing.

## Funding

The authors have nothing to report.

## Ethics Statement

The research was approved by the Bayburt University Research Ethics Committee (07.02.2023/ Decision no: 33). The participants were fully informed about the study, and both written and verbal informed consent were obtained from all participants in accordance with the Declaration of Helsinki before data and samples were collected. All methods were carried out in accordance with the relevant guidelines and regulations.

## Conflicts of Interest

No potential conflict of interest was reported by the authors.

## Independence from Related Publications

This manuscript reports the first author's doctoral‐dissertation dataset collected under Bayburt University Research Ethics Committee approval dated February 7, 2023 (Decision No. 33). No participants, saliva samples or aliquots, assay measurements, raw absorbance values, calculated concentrations, or analytical data records overlap with References 40–43. The present manuscript is not a reanalysis, subgroup analysis, or segmented report of any previously published dataset.

## Supporting information




**Supplementary Material**: brb371674‐sup‐0001‐SuppMat.docx

**Supplementary Tables**: brb371674‐sup‐0002‐TableS1‐S2.docx

## Data Availability

The corresponding author upon reasonable request will provide data supporting the findings of this study.
