# Supplementary material for: Recitation Mode Is Associated With Salivary CREB and Cortisol Responses during Qur'anic Recitation
Source: Brain Behav. 2026 Aug 16;16(8):e71674. doi: 10.1002/brb3.71674 (PMC13478787; doi:10.1002/brb3.71674)
Supplement: Supplementary file 1 [file BRB3-16-e71674-s001.docx]

**Supplementary Material**

Supplementary Table S1. Baseline Salivary CREB and Cortisol Values Across Experimental Conditions

| **Condition** | **n** | **Baseline**  **CREB Mean** | **Baseline**  **CREB SD** | **Cortisol Mean (µg/dL)** | **Cortisol SD (µg/dL)** |
| --- | --- | --- | --- | --- | --- |
| Passive Control | 20 | 0.580 | 0.107 | 2.118 | 0.225 |
| Sight-Reading | 30 | 1.149 | 0.477 | 2.020 | 0.146 |
| Memorized Recitation | 30 | 1.345 | 0.582 | 2.119 | 0.175 |

Note. Values represent absolute baseline salivary concentrations measured before the experimental task or passive rest condition. CREB values are reported in ng/mL; cortisol values are reported in µg/dL after conversion from the manufacturer’s µg/L assay output (µg/L ÷ 10 = µg/dL).

Supplementary Table S2. CREB Change-Score Sensitivity Analysis

| **Condition** | **n** | **Mean ΔCREB** | **SD** | **95% CI** |
| --- | --- | --- | --- | --- |
| Passive Control | 20 | 0.043 | 0.038 | 0.025 to 0.061 |
| Sight-Reading | 30 | 0.310 | 0.145 | 0.256 to 0.364 |
| Memorized Recitation | 30 | 0.500 | 0.324 | 0.379 to 0.621 |

Note. ΔCREB = post-task CREB minus baseline CREB, reported in ng/mL. Change-score variances were unequal, Levene’s F(2, 77) = 41.85, p < .001. Welch’s F(2, 42.89) = 71.22, p < .001. Games–Howell comparisons: Memorized Recitation > Passive Control, p < .001; Sight-Reading > Passive Control, p < .001; Memorized Recitation > Sight-Reading, p = .015.
