# Supplementary material for: Recitation Mode Is Associated With Salivary CREB and Cortisol Responses during Qur'anic Recitation
Source: Brain Behav. 2026 Aug 16;16(8):e71674. doi: 10.1002/brb3.71674 (PMC13478787; doi:10.1002/brb3.71674)
Supplement: Supplementary file 2 [file BRB3-16-e71674-s002.docx]

# **Supplementary Material**

**Title:** Mnemonic Retrieval and Gender Modulate Salivary CREB and Cortisol Levels During Scriptural Recitation

**Authors:** Süheyb OKUR, Yusuf TOPYAY, Bülent BAYRAKTAR

### S1. Detailed Description of the Scriptural Texts

To ensure phonetic and cognitive equivalence between the active intervention groups, the following criteria were applied by an academic commission:

- **Sight-Reading Task:** Surah Al-Qamar (Verses 7-16).
- **Memorized Recitation Task:** Surah Al-Adiyat.
- **Equivalency Rationale:** Both texts were matched for articulatory density, specifically the frequency of emphatic letters unique to Arabic and the complexity of phonetic rules (*tajwid*).

### S2. Biochemical Assay Specifications

All salivary biomarkers were quantified using high-sensitivity Sandwich Enzyme-Linked Immunosorbent Assay (ELISA) kits from SinoGeneclon, adhering strictly to manufacturer protocols:

- **Human CREB ELISA Kit:** (Cat.No SG-11988; Assay Range: 0.625 ng/ml – 20 ng/ml; Sensitivity: 0.078 ng/ml).
- **Human Cortisol ELISA Kit:** (Cat.No SG-00111; Sensitivity: 0.02 ug/L).

### S3. Pre-Collection Participant Protocol

To minimize confounding metabolic and diurnal variables, participants followed a rigorous collection protocol:

- **Diurnal Control:** All saliva samples were consistently collected between 08:00 and 09:00 in the morning to control for hormonal fluctuations.
- **Dietary Restrictions:** Participants were strictly instructed to abstain from consuming food or liquids, brushing their teeth, or ingesting biomarker-altering components (red meat, dairy, nuts, and seeds) for at least one hour prior to sample collection.
- **Sample Processing:** Samples were centrifuged at 2000 g for 20 minutes in a refrigerated centrifuge and stored at -80 °C until quantification.

### S4. Psychometric Assessment

The **Spirituality Scale** used to assess trait-level spirituality encompasses seven sub-dimensions:

- **Dimensions:** Spiritual Coping, Transcendence, Spiritual Experience, Search for Meaning, Spiritual Contentment, Connectedness, and Harmony with Nature.
- **Reliability:** While the original validation reported a reliability coefficient of .90, the Cronbach's alpha for the current study sample was calculated to be .89.

### S5. Statistical Strategy and Assumption Testing

- **CREB Analysis:** Preliminary analyses confirmed compliance with parametric assumptions, allowing for the use of a one-way Analysis of Covariance (ANCOVA) with baseline levels as a covariate.
- **Cortisol Analysis:** Since the assumption of homogeneity of regression slopes was violated (p < .001), change scores (Delta M) were utilized.
- **Robust Testing:** Due to a violation of the homogeneity of variances for cortisol change scores (F(2, 77) = 5.24, p = .007), a robust Welch’s one-way ANOVA was implemented, paired with Tamhane’s T2 post-hoc comparisons.
